# Loneliness and social isolation interventions for older adults: a scoping review of reviews

**DOI:** 10.1186/s12889-020-8251-6

**Published:** 2020-02-14

**Authors:** Olujoke A. Fakoya, Noleen K. McCorry, Michael Donnelly

**Affiliations:** Centre of Excellence for Public Health, Centre for Public Health, Queen’s University, Belfast, Northern Ireland

**Keywords:** Loneliness, Lonely - Social isolation - socially isolated - older adults, Aged - ageing population - scoping, Scoping review

## Abstract

**Background:**

Loneliness and social isolation are growing public health concerns in our ageing society. Whilst these experiences occur across the life span, 50% of individuals aged over 60 are at risk of social isolation and one-third will experience some degree of loneliness later in life. The aim of this scoping review was to describe the range of interventions to reduce loneliness and social isolation among older adults that have been evaluated; in terms of intervention conceptualisation, categorisation, and components.

**Methods:**

Three electronic databases (CINAHL, Embase and Medline) were systematically searched for relevant published reviews of interventions for loneliness and social isolation. Inclusion criteria were: review of any type, published in English, a target population of older people and reported data on the categorisation of loneliness and/or social isolation interventions. Data extracted included: categories of interventions and the reasoning underpinning this categorisation. The methodology framework proposed by Arskey and O’Malley and further developed by Levac, et al. was used to guide the scoping review process.

**Results:**

A total of 33 reviews met the inclusion criteria, evaluating a range of interventions targeted at older people residing in the community or institutionalised settings. Authors of reviews included in this paper often used the same terms to categorise different intervention components and many did not provide a clear definition of these terms. There were inconsistent meanings attributed to intervention characteristics. Overall, interventions were commonly categorised on the basis of: 1) group or one-to-one delivery mode, 2) the goal of the intervention, and 3) the intervention type. Several authors replicated the categorisation system used in previous reviews.

**Conclusion:**

Many interventions have been developed to combat loneliness and social isolation among older people. The individuality of the experience of loneliness and isolation may cause difficulty in the delivery of standardised interventions. There is no one-size-fits-all approach to addressing loneliness or social isolation, and hence the need to tailor interventions to suit the needs of individuals, specific groups or the degree of loneliness experienced. Therefore, future research should be aimed at discerning what intervention works for whom, in what particular context and how.

## Background

Loneliness and social isolation are international public health concerns that particularly affect the ageing society globally [1]. Loneliness and social isolation are distinct but interrelated concepts. According to Valtorta and Hanratty [2], one of the most widely used definitions of loneliness constitutes of social and emotional loneliness: loneliness is a subjective negative feeling associated with a perceived lack of a wider social network (social loneliness) or absence of a specific desired companion (emotional loneliness). There is much less consensus about the definition of social isolation however authors have approached it as a multidimensional concept, defining social isolation as the *objective* lack or paucity of social contacts and interactions with family members, friends or the wider community [2].

Loneliness and social isolation are risk factors for all-cause morbidity and mortality with outcomes comparable to other risk factors such as smoking, lack of exercise, obesity and high blood pressure [3–5]. In addition, loneliness has been associated with decreased resistance to infection, cognitive decline and mental health conditions such as depression and dementia [3]. Whilst every individual will experience loneliness at some point in their lives to a certain degree [6], research has highlighted that older people are particularly vulnerable to experiencing loneliness and social isolation [7, 8]. Approximately 50% of individuals aged over 60 are at risk of social isolation and one-third will experience some degree of loneliness later in life [3]. Although loneliness and social isolation have been associated with a reduction in health status and therefore a decreased quality of life, findings suggest that both concepts may have independent impacts on health and therefore should be regarded as individual characteristics [9]. However, there is also an overlap in the factors which contribute to loneliness and social isolation and sometimes authors use the terms interchangeably [10, 11].

Risk factors for loneliness and social isolation among older people include: family dispersal, decreased mobility and income, loss of loved ones, and poor health. It is thought that societal change including reduced inter-generational living, greater geographical mobility and less cohesive communities have also contributed to higher levels of loneliness in the older population [7, 12]. Due to advancements in public health and medical technologies, in addition to improved sanitation, the average life expectancy of the population aged 60 years or over has increased globally, resulting in a projected 56% growth in this population from 901 million to 1.4 billion by 2030 [13]. Healthy life expectancy however still lags behind, and the increasing prevalence of loneliness contributes to this state of affairs [14].

Given the increasing burden of loneliness and its impact on health and wellbeing, it is not surprising that there has been a growing academic literature, public and policy interest worldwide in loneliness and social isolation. For example, the Campaign to End Loneliness began in 2010 in the United Kingdom (UK) and aimed to create connections among older age people [8]. In Denmark, a campaign titled ‘*Danmark spiser sammen*’ which when translated in English means ‘Denmark eats together’ was established in 2015 as a popular movement against loneliness [15]. The Australian Coalition to End Loneliness (ACEL), inspired by the Campaign to End Loneliness in the UK, was developed in Australia in 2016 and aimed to use evidence-based interventions and advocacy to increase awareness of, and address, loneliness and physical social isolation [16]. ACEL did not clarify what was meant by the term ‘physical social isolation’ and this further highlights the varied terminology used regarding loneliness and social isolation. There are also growing campaigns in the Netherlands and New Zealand to tackle loneliness [1]. ALONE, a national organisation in Ireland that offers support to older people, launched a Christmas campaign in 2018 called ‘Have a Laugh for Loneliness’ which encouraged families, friends and communities to get together during the winter in order to combat loneliness in their communities [17].

Several reports about the range and types of loneliness interventions have been published globally. Within the United Kingdom, these have included reports by organisations such as Age UK [18] and the Institute of Public Health in Ireland [19]; guidelines by the National Institute for Clinical Excellence [20]; reviews by the Social Care Institute for Excellence [7, 21], and material collated by the Campaign to End Loneliness [1]. The Canadian Counselling and Psychotherapy Association (CCPA) have published guidelines for addressing loneliness [22]. Similarly, in the United States of America (USA), organisations such as Humana [23], have published reports and a toolkit to overcome loneliness and social isolation, and the National Institute on Aging (National Institutes of Health) [24] have published reports on improving the development of interventions to reduce loneliness and social isolation.

The report published by Age UK [25] specifically highlighted the gap between evidence of what constitutes an effective ‘loneliness intervention’ in the academic literature and the practice of those delivering interventions. Nevertheless, service providers are experiencing increasing demand to provide initiatives to tackle loneliness, even in the absence of empirical evidence to fully support their innovations.

There are several published systematic reviews of loneliness and/or social isolation interventions, e.g. Cattan and White [26], Cattan, et al. [10] and Dickens, et al. [9]. For example, Cattan and White [26] critically reviewed the evidence of effectiveness of health promotion interventions targeting social isolation and loneliness among older people. It was reported that an effective intervention to combat social isolation and loneliness among older people tended to be long-term group activity aimed at a specific target group, with an element of participant control using a multi-faceted approach [26]. Cattan, et al. [10] conducted a systematic review to determine the effectiveness of health promotion interventions that targeted social isolation and loneliness among older people, and found educational and social activity interventions that target specific groups can alleviate social isolation and loneliness among older people. However, the effectiveness of home visiting and befriending schemes remains unclear [10]. Similarly, a systematic review conducted by Dickens, et al. [9] aimed to assess the effectiveness of interventions designed to alleviate social isolation and loneliness in older people. It was reported that common characteristics of effective interventions were those developed within the context of a theoretical basis, and those offering social activity and/or support within a group format. Interventions where older people were active participants also appeared more likely to be effective [9].

Within this diverse literature, there are a range of frameworks used to categorise loneliness/social isolation interventions, often without clear definitions or rationale. Hence, there is a need to: map, organise and synthesise the large and diverse body of literature in this area; describe the range of intervention types; and to synthesise their content and characteristics.

Scoping reviews are useful for synthesising research evidence and are often used to categorise existing literature in a field. They can be used to map literature in terms of nature, features and volume; to clarify definitions and conceptual boundaries; and to identify research gaps and recommendations. They are particularly useful when a body of literature exhibits a large, complex or heterogeneous nature [27].

### Scoping review objectives

The objective of this scoping review is to map the large body of literature and to describe the range of interventions to reduce loneliness and social isolation among older adults. By focusing on existing reviews of loneliness/social isolation interventions, it aims to synthesise the ways in which interventions have been conceptualised and their components described.

### Scoping review questions

How have authors of the reviews that were included in this paper (hereafter referred to as ‘review authors’) grouped or categorised loneliness and social isolation interventions?

How have review authors defined the terms used to categorise interventions?

How have review authors described their reasoning for categorising interventions in the format used?

Are there any similarities or differences in the terms used to categorise interventions across the reviews?

## Methods

The conduct of this scoping review was based on the framework and principles reported by Arksey and O’Malley [28] and further recommendations provided by Levac, et al. [29]. Additional guidance on reporting by Peters, et al. [27] was also used. As the primary interest was in capturing how loneliness and social isolation interventions are categorised and described in the literature, an efficient way of doing this was to focus on review papers (of any type) rather than primary literature. Appropriate adjustments were made to reflect the nature of the evidence (i.e. only secondary evidence) being reviewed. The review included the following 5 key phases [28]:

- Stage 1: Identifying the research question
- Stage 2: Identifying relevant studies
- Stage 3: Study selection
- Stage 4: Charting the data
- Stage 5: Collating, summarising and reporting the results

The optional ‘consultation exercise’ recommended by Arskey and O’Malley [28] was not conducted.

### Information sources and search strategy

Following several preliminary scoping searches which were intended to gain familiarity with the literature and aid with the identification of key words, three health bibliographic databases (Medline, EMBASE and Cumulative Index to Nursing and Allied Health Literature (CINAHL)) were searched for relevant literature from their inception until the date that the search was conducted (15th June 2018). Searches were devised in collaboration with an information specialist librarian and the research team. The search strategy was developed to identify reviews of loneliness/social isolation interventions for older people, but the strategy was tailored to the specific requirements of each database as seen in Additional file 1: Table S1. Grey literature was searched using Google (including Google Scholar) and the first 30 links (sorted by relevance) were compared against the inclusion criteria. Backward citation chaining was also undertaken which involved hand-searching the reference lists of the reviews identified to find other relevant research [30]. Electronic search results were exported into an Excel spreadsheet and duplicates deleted. Additional file 1: Table S1 details the search terms and strategy.

### Eligibility criteria

Whilst loneliness and social isolation are distinct concepts (as defined previously), we have included both outcomes as a focus of the review but have taken care to document the review findings in relation to these concepts. Hence, papers were included if they satisfied all of the following eligibility criteria:

1. A review of any type;
2. Available in English language;
3. Focus of the review on loneliness and/or social isolation interventions for older adults/elderly individuals;
4. Reported a categorisation of loneliness and/or social isolation interventions or grouped interventions.

Reviews of interventions in any setting or context, including older populations with existing physical or mental health problems were of interest. Since there are various definitions of the age range of ‘older’ populations, a lower age limit was not specified as an inclusion criteria. Rather, reviews were included which identified *themselves* as focusing on older people. There were no limiters applied in relation to date or subject, but the search was limited to reviews published in English because of limited resources for translation.

### Selection of reviews

**Fig. 1:**
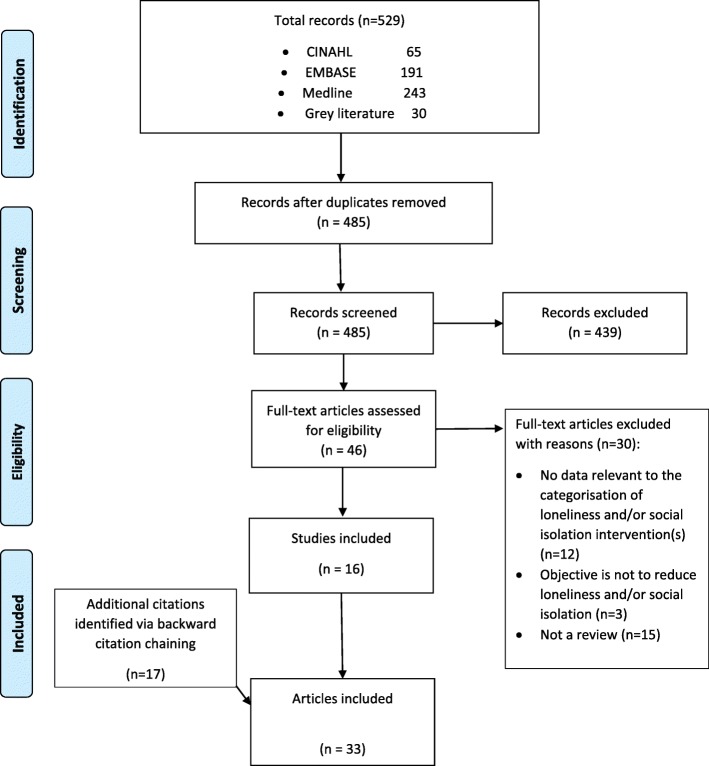
PRISMA flow diagram illustrating the search strategy. This flow diagram provides the phases of article identification and selection, which resulted in the identification of 33 articles that were deemed eligible for inclusion in the review. Prepared in accordance with Tricco AC, et al. PRISMA Extension for Scoping Reviews (PRISMA-ScR): Checklist and Explanation. Annals of Internal Medicine. 2018. pp. 467–473. doi: 10.7326/M18-0850

The selection of relevant reviews was undertaken in three stages: 1) Initial screening of the title and abstract which was conducted by the first author (OAF), 2) retrieval and screening of the full text which was completed independently by the first and second authors (OAF and NMC), with discrepancies resolved through discussion with all three authors, and 3) data extraction and collation. The agreement coefficient was 97%. Papers that did not meet the criteria were excluded, with the reason(s) for exclusion recorded. The Preferred Reporting Items for Systematic Reviews and Meta-Analysis (PRISMA) chart (Fig. 1) reports the phases of paper identification and selection.

### Data extraction and charting

Eligible papers were independently reviewed by OAF and NMC and the following data were extracted: author information (title, author and year of publication), aims and objectives of the review, type of review (e.g. systematic, literature etc.), inclusion criteria used in the review (where appropriate), number of primary studies included in the review (where appropriate), number of interventions reviewed (where appropriate), categories used by the review authors, and any explanation given by the authors in regards to the categorisation of interventions. It should be noted that the following parameters were not applicable to non-systematic type reviews such as basic literature reviews and some evidence reviews: inclusion criteria; number of primary studies; and number of interventions reviewed.

## Results

### Literature search

Electronic searches identified 529 citations, resulting in 485 unique citations to be screened for inclusion following removal of duplicates (see Fig. 1). The titles and abstracts were assessed for their relevance to the review based on the inclusion criteria (Stage 1 screening), resulting in 46 citations being retained. The full texts of all these citations were obtained and after applying the inclusion criteria (Stage 2 selection), 30 citations were excluded; 12 did not provide data relevant to categorisation of loneliness and/or social isolation intervention(s), 15 were not reviews and three did not have a primary or secondary objective of reducing loneliness and/or social isolation. An additional 17 citations were identified through backward citation chaining and these citations were also included. As such, 33 citations were included in the scoping review (see Fig. 1). Characteristics of the included reviews are shown as a structured table and as a narrative summary in Additional file 2: Table S2.

### Characteristics of reviews

**Fig. 2:**
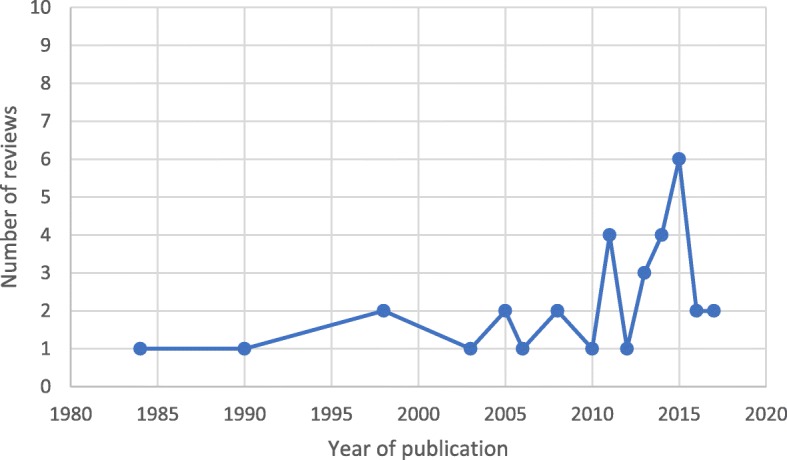
Number of review articles on loneliness and social isolation interventions published from 1984 to 2018. The first review of loneliness and social isolation interventions appeared in 1984 and following that, there were three more reviews up until the year 2003. There were more frequent publications of reviews on loneliness and/or social isolation from 2010 onwards

There is increasing interest and research in the area of loneliness and social isolation among the older population. The first review appeared in 1984 and following that, there were three more reviews up until the year 2003. Subsequently, there were more frequent publications of literature on loneliness and/or social isolation and at least one review was published consecutively every year from 2010 onwards. This information is represented in a diagrammatic form in Fig. 2.

#### Type of reviews

**Fig. 3:**
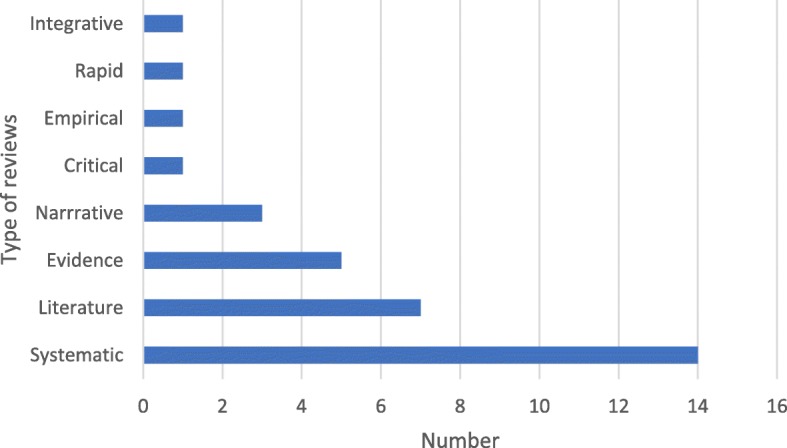
Type of review articles on loneliness and social isolation interventions published from 1984 to 2018. Systematic reviews were the most common type of reviews published between 1984 and 2018. Other types of reviews include literature, evidence, narrative, critical, empirical, rapid and integrative reviews

Review papers were published between 1984 and 2017 and of these, systematic reviews were the most common type of reviews obtained [9, 10, 26, 31–41], followed by literature reviews [6, 42–47], evidence reviews [18, 48–50], narrative reviews [25, 51, 52], and other types of review including critical [53], empirical [54], rapid [55] and integrative review [11]. This information is represented in a diagrammatic form in Fig. 3.

Of those reviews which employed a systematic means of selecting eligible primary research (*n* = 14), ten papers included only studies published in English, two review papers included studies published in any language, one review included studies published in English and Italian [34], and 1 included studies published in English, French, Italian and Spanish [41].

#### Concept of loneliness and social isolation

In terms of the consideration of the concepts of loneliness and social isolation, most reviews (28/33) could be assigned to one of three categories: 1) reviews that explicitly focused on interventions to reduce social isolation (*n* = 4) e.g. Chen and Schulz [37], Findlay [33], Oliver, et al. [47] and Wilson and Cordier [52]; 2) reviews that explicitly focused on interventions to alleviate loneliness (*n* = 11), e.g. McWhirter [6] and Masi, et al. [31] and Cohen-Mansfield and Perach [53]; and 3) reviews that included papers with interventions for both loneliness and social isolation (*n* = 13) e.g. Poscia, et al. [34] and Cattan, et al. [10]. The remaining five reviews focused on loneliness and other outcomes of interests such as anxiety and depression (*n* = 3); or other related concepts such as social participation [56], and social connectedness [35]. While there is a distinction between loneliness and social isolation, there was not any obvious differences in reviews that focused on loneliness or social isolation in terms of the review type, where the research was conducted, and how the findings were reported.

Loneliness/social isolation was not always reported as the primary outcome and was sometimes reported alongside other health outcomes as seen in three reviews [36, 38, 40]. A review by Choi, et al. [40] examined the effectiveness of computer and internet training on reducing loneliness and depression in older adults. Elias, et al. [38] evaluated the effectiveness of group reminiscence therapy for loneliness, anxiety and depression in older adults. In a review by Franck, et al. [36], interventions were reviewed if they addressed social isolation, loneliness, or the combination of depression with social isolation or loneliness. In a systematic review by Morris, et al. [35], the effectiveness of smart technologies was examined in improving or maintaining social connectedness.

#### Population characteristics

The majority of the reviews (*n* = 24) focused solely on the older population [9–11, 18, 25, 26, 32–41, 46, 48–51, 53, 55, 56] but the age range used to define this population varied [32, 35–38, 53], or was not specified at all [9–11, 26, 33, 46, 51, 56]. For example, a systematic review by Morris, et al. [35] targeted older people who live at home and included participants that were aged ≥45 years, whereas Cohen-Mansfield and Perach [53] and Chen and Schulz [37] targeted individuals aged ≥55 years; and Chipps, et al. [32], Franck, et al. [36], and Elias, et al. [38] targeted individuals aged ≥60 years. Where age was not specified, review authors used the term ‘older people’ or its synonyms, e.g. older adults [40] and seniors [39, 56], to describe the target population. It was stated in two of these reviews that the definition for the older person was defined by the criteria used in the studies included in the review [26, 56].

**Fig. 4:**
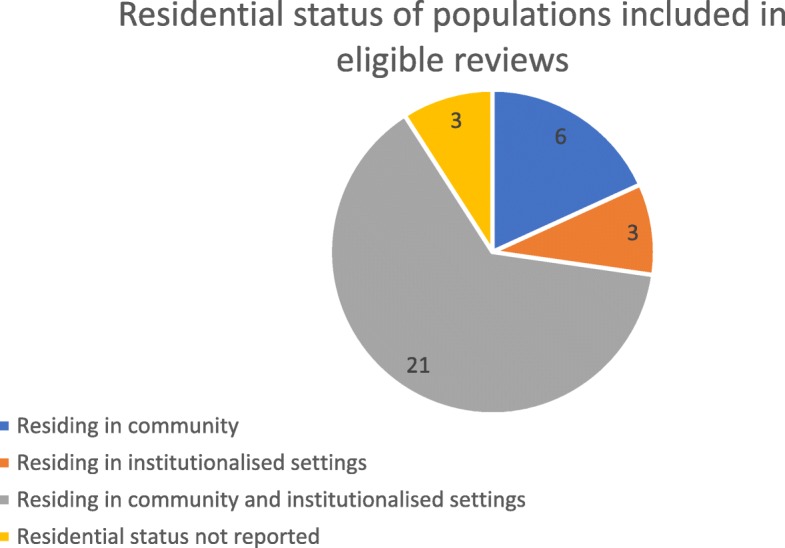
Pie-chart of residential status of populations included in eligible reviews identified. The majority of reviews (*n* = 21) included populations of both community-dwelling individuals and those living in long-term care. Six reviews focused solely on older people residing within the community and three focused solely on older people living in institutionalised settings such as care or nursing homes. Three reviews did not report the residential status of the target population

Some reviews focused on specific subgroups of the older population which research has identified to be more prone to loneliness and social isolation. For example, six reviews focused only on older people residing within the community [6, 10, 39, 42, 48, 52], whereas three focused only on older people living in institutionalised settings e.g. care or nursing homes [36, 38, 47]. The majority of reviews (21 in total) included populations of both community-dwelling individuals and those living in long-term care [9, 11, 18, 25, 31–35, 37, 40, 41, 44–46, 49–51, 53, 55, 56]. Residential status was not reported in three reviews [26, 43, 54]. This population characteristic is represented diagrammatically in Fig. 4.

Only two of the 33 reviews [41, 52] included gender as an inclusion criteria, and these two papers focused specifically on interventions targeted at the male population only, including Men’s Sheds [52] and gendered interventions for older men [41]. Men’s Sheds are community-based organisations that provide a space for older men to participate in craftwork and engage in social interaction [52]. Review authors often reported that the gender distribution of participants in primary research involving loneliness/social isolation was heavily skewed towards the female population [31, 33, 34, 37, 39]. The subsets of the female population reported in the reviews includes: isolated older women, women at risk of suicide, senior women on the housing waiting list [33]; community-living, chronically ill women [31]; women with primary breast cancer, community-dwelling, single women [34]; and community-dwelling low-income women with low perceived social support [39].

#### Countries in which interventions were delivered

The countries in which interventions were delivered was not reported in some of the review papers (*n* = 16). Of the papers that did report this (*n* = 17), USA was the most reported (*n* = 14), followed by Netherlands (*n* = 13), Canada (*n* = 10), UK (*n* = 9), Australia (*n* = 8), Sweden (n = 8), Finland (*n* = 5), Taiwan (n = 5), Israel (*n* = 4), Norway (n = 4), Germany (n = 4), Japan (*n* = 3), China (*n* = 2), Hong Kong (n-2), Denmark (n = 2), Italy (n = 2), New Zealand (n = 2), South Africa (n = 1), Austria (n = 1), Slovenia (n = 1) and Iran (n = 1).

#### Categorisation of interventions

There was a broad range of terms that review authors used to describe the characteristics of interventions, such as: format [31], delivery mode [9, 31, 34], goal [42, 44, 46], type [9, 31, 34, 53], focus [53], and nature [46], and often the same terms had different meanings. Some authors used two or more categorisation systems as seen in the reviews by Dickens, et al. [9] and Poscia, et al. [34], where interventions were categorised by both their ‘delivery mode’ and ‘type’. Alternatively Grenade and Boldy [46] categorised interventions by their ‘nature’ and ‘goal’, and Cohen-Mansfield and Perach [53] categorised interventions based on their ‘focus’ and ‘type’. Masi, et al. [31] categorised interventions based on their ‘type, format and mode’. It was common (*n* = 20) for review authors to categorise interventions on the basis of whether they were delivered via a ‘group’ or ‘one-to-one’ [9, 10, 18, 26, 31, 33, 34, 36, 38, 39, 42, 44–46, 49–51, 53, 55, 56]. In a review by Raymond, et al. [56], social participation interventions were delivered in an individual or group context. Elias, et al. [38] explored the effectiveness of group reminiscence therapy in alleviating loneliness whereas the Medical Advisory Secretariat [39] evaluated in-person group-based interventions in alleviating loneliness and social isolation among community-dwelling care seniors. The term ‘mode’ was used frequently within review papers but often with inconsistent meanings. By way of illustration, Poscia, et al. [34] and Dickens, et al. [9] referred to the categorisation of interventions via group or one-to-one delivery as ‘mode’, and classified interventions as individual, group or mixed (both individual and group). In contrast, delivery ‘mode’ in Masi, et al.’s [31] review referred to ‘technology’ or ‘non-technology’ based interventions, and ‘format’ was used to describe whether the intervention was implemented on a one-to-one basis or as a group (if more than one person participated in the intervention at the same time or if the intervention involved asynchronous interactions such as internet-based chat room exchanges).

Some review authors categorised interventions by their type (*n* = 4) [9, 31, 34, 53], and the descriptions for this category also varied. In a review by Dickens, et al. [9], interventions categorised by their ‘type’ were described as: ‘offering activities’ (e.g. social or physical programmes), ‘support’ (discussion, counselling, therapy or education), ‘internet training’, ‘home visiting’ or ‘service provision’. In another review, intervention type was described as: 1) social skills training if it focused on improving participants’ interpersonal communication skills, 2) enhancing social support if the intervention offered regular contacts, care or companionship, 3) social access if the intervention increased opportunities for participants to engage in social interaction (e.g. online chat room or social activities), and 4) social cognitive training if the intervention focused on changing participants’ social cognition [31].

Similar to the categories used in the review by Masi, et al. [31], Poscia, et al. [34] categorised interventions by their type, further describing the category as offering: [1] social support (e.g. discussion, counselling, therapy or education), 2) social activities, in form of social programmes, 3) Physical activity (fitness programme or recreational activity), 4) technology (e.g. companion robot, telephone befriending or internet use), 5) singing sessions, and 6) horticultural therapy. By contrast, when Cohen-Mansfield and Perach [53] categorised interventions by their ‘type’ this referred to whether interventions were delivered in a ‘group’ or ‘one-to-one’.

Three reviews categorised interventions by their ‘goal’ [42, 44, 46]. In two of these reviews [42, 44] the same constructs were used to define goals and these were: 1) to facilitate social bonding e.g. via cognitive behaviour therapy or social skills training, 2) to enhance coping with loneliness e.g. through support groups, and 3) to prevent loneliness from occurring e.g. through community awareness and educational programs. In the third review [46], the authors *implicitly* addressed these three constructs but used different terminology, i.e. to enhance people’s social networks, and promote personal efficacy and behaviour modification, and/or skills development. A similar categorisation system was used by Cacioppo, et al. [45], but these review authors labelled this category as ‘models of loneliness interventions’ rather than ‘goal’, and included interventions aimed to: 1) provide social support, 2) increase opportunities for social interaction and 3) teach lonely people to master social skills.

A total of six reviews focused on technology-based interventions to improve communication and social connection among older people [32, 35, 37, 40, 47, 48]. An evidence review by Age UK [48] reviewed the use of modern (e.g. internet) and assistive technology (e.g. telecare or telehealth) in maintaining and establishing social contact. Chen and Schulz [37] reviewed the effects of communication programs such as telephone befriending, computer and internet, and high-technology apps such as virtual pet companions in reducing loneliness and social isolation in the elderly. The effectiveness of e-interventions which can be described as online activities e.g. computer or internet training and usage; interpersonal communication e.g. Skype; and internet-operated therapeutic software e.g. Nintendo Wii entertainment system and videogames, were synthesised and assessed for decreasing social isolation and loneliness among older people living in community/residential care [32]. One systematic review evaluated the effectiveness of smart technologies [35], which can be described as internet-based support groups and computer use and training, whereas the potential of videophone technology in improving communication between residents and family members was reviewed by Oliver, et al. [47]. In another review, computer and internet training among lonely and depressed older adults were examined [40].

The rationale for the categorisation of interventions was reported in the majority of reviews (*n* = 21). It was stated in an integrative review by Gardiner, et al. [11] that interventions were categorised based on their purpose, intended outcomes and mechanisms by which they targeted loneliness and social isolation. Gardiner, et al. [11] highlighted the importance of this categorisation given the growing diversity in intervention types, and considered rigorous and transparent categorisation to be a necessary pre-requisite for identifying which elements of interventions influence their effectiveness. Their thematic synthesis identified six categories which included: social facilitation interventions, psychological therapies, health and social care provision, animal interventions, befriending interventions, and leisure/skills development. In a narrative synthesis by Jopling [25], interventions were grouped in accordance to addressing three key challenges: 1) reaching lonely individuals, 2) understanding the nature of an individual’s loneliness and developing a personalised response, and 3) supporting lonely individuals to access appropriate services.

Other reviews [36–41, 47–51, 55] did not report a rationale for the categorisation of interventions (as seen in Additional file 2: Table S2). Some review authors justified their categories on the basis that they had been used in previous reviews, e.g. two reviews [10, 33] replicated the categorisation used in a previous study by Cattan and White where intervention studies were divided into four categories based on the programme or method type, i.e. group activity; one-to-one intervention; service delivery; and whole community approach [26]. Likewise, McWhirter [6] used similar categories as Rook and Peplau [57], such as cognitive-behavioural therapy, social skills training, and the development of social support networks; Andersson [42] categorised interventions based on the typology of social network interventions by Biegel, et al. [58] (either clinical treatment, family caretaker enhancement, case management, neighbourhood helping, volunteering linking, mutual aid/self-help, and community empowerment); and Masi, et al. [31] categorised the intervention type (i.e. providing social access, social cognitive training, social skills training or social support) based on similar constructs used in the reviews by Rook [44], McWhirter [6], Cattan and White [26], Findlay [33], Cattan, et al. [10] and Perese and Wolf [43].

## Discussion

The objective of this scoping review was to map the large body of literature and describe the range of interventions aimed at reducing loneliness and/or social isolation among older adults. By focusing on existing reviews of loneliness/social isolation interventions, it aimed to synthesise the ways in which interventions have been conceptualised and their components described.

There are various interpretations of loneliness and social isolation in the literature. Social isolation can be defined as ‘a state in which an individual lacks a sense of belonging socially, lacks engagement with others, and has a minimal number of social contacts which are deficient in fulfilling quality relationships’ [59–62]. On the other hand, loneliness can be defined as a ‘subjective state based on a person’s emotional perception of the number and/or quality of social connections needed in comparison to what is being experienced at the time’ [63, 64]. There is evidence to suggest that both concepts are distinct [9, 65–67] as an individual can have a large number of social connections and still experience the subjective feeling of loneliness, or alternatively be objectively isolated but not experience loneliness [68]. For some individuals, social isolation is a risk factor for loneliness [18], and hence interventions designed to target social isolation may also alleviate loneliness. For other individuals, where the pathway to loneliness is not as a result of social isolation, such interventions are likely to have limited impact.

Although it is generally understood that loneliness and social isolation are distinct concepts, some review authors have stated that the terms are often used interchangeably [10, 11, 46] or are conflated into a single construct [68]. While there were fewer reviews identified that specifically focused on social isolation (*n* = 4) compared to loneliness (*n* = 11), there were no differences in terms of the countries where the research was conducted, the review type, or how the findings were reported. Distinguishing between the concepts of loneliness and social isolation is important when describing the goals of interventions and hence for specifying intervention characteristics that are relevant and effective in addressing each of these problems [4]. This clarity is necessary if service providers are to use the accumulated evidence to choose interventions which are appropriate and effective relative to their service context and goals, for matching individuals to appropriate interventions, and for choosing appropriate outcome measures for evaluation. Rook [44] made reference to the causes of loneliness and often linked these with the ‘goal’ of the interventions. Social inhibition or deficient social skills were linked to loneliness for some people and hence it was suggested that helping lonely individuals establish interpersonal ties might improve how they relate to others or provide new opportunities for them to have social contact. Alternatively, in circumstances where an individual was geographically isolated, an intervention which improves the social network may be more appropriate.

Review authors have used a range of terms to categorise the characteristics of interventions, such as mode of delivery, focus, nature, format, type and goal, but often with different meanings. Interventions were commonly categorised only by whether they were delivered to a group or to an individual. This is an important characteristic because group interventions are likely to be more appropriate for addressing social loneliness among individuals with insufficient social links [69] than one-to-one interventions. However, it is only one of many intervention characteristics which may be directly, or via interaction with other characteristics, associated with intervention effectiveness.

Terms and terminology are important when undertaking research in the field of loneliness [70]. Consistency in the definition of the terms and terminology increases accuracy, improves reporting, and aids in the replication of interventions across contexts [71].

In some reviews, the underlying theoretical basis or rationale for the categorisation of interventions was not provided. Lack of theoretical underpinnings or explanations as to why interventions were categorised in a certain manner could lead to difficulty when attempting to distinguish in what context a particular category of intervention is most appropriate or effective. This reduces the value of the accumulated evidence base, since we are less able to identify candidate characteristics that may contribute to the effectiveness of interventions. Hence, there is a need for the development of a comprehensive framework that encompasses, defines, and elucidates all the key constructs identified in this scoping review. Without this framework, research to identify the effective mechanisms of loneliness interventions will be undermined by lack of clarity around intervention characteristics.

Interventions to reduce loneliness and/or social isolation are complex as they have several interacting components (e.g. goals, personnel, activities, resources and delivery mode), which may interact with features of the local context in which they are applied (e.g. age profile of participants, health status, environment such as housing, and cultural characteristics) [72]. These characteristics need to be sufficiently described in order to allow use of the body of evidence to identify which characteristics (or combination of characteristics) are effective in a particular context and for which specific population.

The Template for Intervention Description and Replication (TIDieR) checklist and guide, published by Hoffmann, et al. [73] was developed as an extension of the Consolidated Standards of Reporting Trials (CONSORT) 2010 statement [74] and the Standard Protocol Items: Recommendations for Interventional Trials (SPIRIT) 2013 statement [75]. The TIDieR checklist provides a standardised template for authors to describe key elements for reporting of non-pharmacological interventions. The development of the checklist is associated with a wider movement towards standardising research reporting, demonstrated by the growing EQUATOR (Enhancing the QUAlity and Transparency Of health Research) network [73]. The overarching purpose of the TIDieR checklist is to prompt authors to describe interventions sufficiently in order to allow their replication [73].

The benefits of using the TIDieR framework is that it can be used for better description and reporting of interventions. This may lead to a more standardised reporting of intervention characteristics particularly in the primary literature, and therefore make synthesis of the literature more consistent. Additionally, it allows for comparison of key characteristics of interventions and for synthesis of interventions that share similar characteristics. The checklist makes it easier for authors to structure the accounts of their interventions/services; for editors to assess these descriptions; and for readers to use the information [73]. However, although the TIDieR checklist may go some way towards assisting with the reporting of complex interventions, it might not be able to capture the full complexity of these interventions [73] such as the interaction between different intervention components or their combined effect, the difficulty or complexity of behaviours/skills required either by those delivering or receiving the intervention; and also variability of outcomes [76]. This is particularly relevant to loneliness/social isolation interventions which rely on more than one mechanism, therefore making it unclear which particular aspect of the intervention contributed most to its success or failure.

The heterogeneous nature of the interventions aimed at alleviating loneliness and/or social isolation among the older population; the settings where they are delivered e.g. care home or community; the group or one-to-one intervention delivery mode; and the population characteristics described in this scoping review, present a challenge for policy recommendations. The individuality of the experience of loneliness is also an important issue which has also been highlighted in the literature, as this may cause difficulty in the delivery of standardised interventions [3]. There is no one-size-fits-all approach to loneliness interventions [25, 70], and it is recommended that the assessment of individual needs should be conducted during the early phases of intervention, with subsequent tailoring of programmes to meet the needs of individuals [77], specific groups or the degree and determinants of the individual’s loneliness. This includes sociodemographic factors i.e. age, poverty, being a carer; the social environment i.e. access to transport, driving status and place or resident; and physical or mental health [2]. It is also essential to consider the needs of less well-researched groups such as individuals with physical disabilities, or ethnic minority groups, caregivers, recent immigrants, individuals with hearing and visual impairments, those who have been isolated for a long time, and older men [78]. Several review authors have reported that the uptake of participants in the primary studies was heavily skewed towards the female population. This may be due to the reluctance of older men to engage with services and activities compared to women [41]. Moreover, women also have a longer life expectancy across nations than men, and are more likely to participate in research studies [37].

Systematic reviews are most appropriate for synthesising the findings of research that evaluates clinical treatments (simple interventions) [79] and consequently base their estimates of effectiveness on one (or more) of the intervention characteristics, e.g. group or individual delivery settings. Complex interventions have several interconnecting parts and it is recognised that the evaluation of this type of interventions should go beyond the question of effectiveness to identify ‘mechanisms’ of action which can be described as the resources offered through an intervention and the way that people respond to those resources (for example, how do resources intersect with participant’s beliefs, reasoning, attitude, ideas and opportunities?) [80, 81]. Hence, a realist review may be a more suitable approach to research synthesis when attempting to understand the mechanisms by which complex social interventions work (or not) in particular contexts [62]. The realist review is a model of research synthesis that is designed to work with complex interventions or programmes and provides an *explanatory* analysis aimed at discerning what works, for whom, in what circumstances, in what respects and how [82]. This approach is more likely to result in findings that will help to identify and tailor interventions to fit the profile of the individual and their pathway to loneliness.

## Strengths and limitations

A strength of this scoping review is that it is the first review of its type to examine the range of loneliness interventions for the older population and to describe how these interventions have been reported and categorised. It has highlighted the need for an appropriate framework to specify and describe the nature of loneliness and social isolation interventions, ideally a framework which defines interventions based on their mechanisms of action, and as a result helps to tailor or choose interventions which are matched to the individual’s needs and pathway to loneliness. Although this review utilised multiple databases and grey literature, searching other databases such as Cochrane Library and PsychInfo may have yielded other relevant published papers relevant to the aims of this scoping review. In addition, because the review was limited to papers published in the English language, it is possible that other potentially relevant reviews were omitted. A quality assessment of the reviews included was not undertaken, although this is not always necessary for scoping reviews (Arksey and O’Malley, 2006).

## Conclusion

A broad range of interventions have been developed in an attempt to combat loneliness and social isolation among older people. Interventions were often categorised solely on the basis of whether they were delivered to a group or an individual. Moreover, the underlying theoretical basis or rationale for the categorisation was not provided in a third of reviews. Lack of theoretical reasoning could lead to difficulty when attempting to distinguish in what context a particular category of intervention is most appropriate or effective, and also by which mechanisms these interventions work to reduce loneliness and social isolation. Comprehensive description of these interventions, using appropriate and consistent terminology should be encouraged as this will increase the value of the accumulated evidence base for service providers and policy-makers. Not all older people experience loneliness in the same way or to the same degree and hence there is a pressing need to tailor interventions to meet individual’s requirements. It is recommended that future research differentiates the diverse group of older adults and takes an approach aimed at discerning what interventions work for specific subsets of this population; the contexts where these interventions work; and the mechanisms by which they operate in that given context. This information will be highly valuable in the planning and implementation of programmes to reduce loneliness and social isolation, and improving the wellbeing of older people.

## Supplementary information

**Additional file 1: Table S1.** Scoping review search strategies.

**Additional file 2: Table S2.** Characteristics of reviews included in the scoping review.

## Abbreviations

ACEL: Australian Coalition to End Loneliness
CCPA: Canadian Counselling and Psychotherapy Association
CINAHL: Cumulative Index to nursing and allied health literature
CONSORT: Consolidated standards of reporting trials
EQUATOR: Enhancing the QUAlity and Transparency Of health Research
ICT: Information communication technology
PRISMA: Preferred reporting items for systematic reviews and meta-analysis
RCT: Randomised controlled trial
SPIRIT: Standard protocol items: recommendations for interventional trials
TIDieR: Template for intervention description and replication
UK: United Kingdom
USA: United States of America
VC: Videoconferencing
