# Supplementary material for: Loneliness and social isolation interventions for older adults: a scoping review of reviews
Source: BMC Public Health. 2020 Feb 14;20:129. doi: 10.1186/s12889-020-8251-6 (PMC7020371; doi:10.1186/s12889-020-8251-6)
Supplement: Supplementary file 1 — Additional file 1: Table S1. Scoping review search strategies. [file 12889_2020_8251_MOESM1_ESM.docx]

**Table 1: Scoping review search strategies**

CINAHL search strategy (Keywords: North American Nursing Diagnosis Association (NANDA); Clinical Care Classification system (CCC); University of California, Los Angeles (UCLA)

| **#** | **Query** | **Results** |
| --- | --- | --- |
| S6 | S3 AND S4 | 65 |
| S5 | S3 AND S4 | 66 |
| S4 | (MH ‘‘Aged, 80 and Over’’) OR (MH ‘‘Frail Elderly’’) | 9,598 |
| S3 | S1 OR S2 | 7,866 |
| S2 | (MH ‘‘Social Isolation’’) OR (MH ‘‘Risk for Loneliness (NANDA)’’) OR (MH ‘‘Social Isolation (NANDA)’’) OR (MH ‘‘Social Isolation (Saba CCC)’’) | 5,699 |
| S1 | (MH ‘‘Loneliness’’) | 2,577 |

EMBASE (Ovid) search strategy (Keyword: University of California, Los Angeles (UCLA).

| **#** | **Searches** | **Results** |
| --- | --- | --- |
| 1 | UCLA Loneliness Scale/ or loneliness/ | 6052 |
| 2 | social isolation/ | 20437 |
| 3 | 1 or 2 | 25546 |
| 4 | limit 3 to English language | 22977 |
| 5 | limit 4 to human | 18978 |
| 6 | limit 5 to ‘‘review’’ | 2024 |
| 7 | limit 6 to aged <65+ years> | 191 |

Medline (Ovid) search strategy

| **#** | **Searches** | **Results** |
| --- | --- | --- |
| 1 | Loneliness/ | 2918 |
| 2 | Social isolation/ | 12193 |
| 3 | 1 or 2 | 14498 |
| 4 | Middle Aged/ or ‘‘Aged, 80 and over’’/ or Aged/ or Aging/ or Adult/ | 6502741 |
| 5 | 3 and 4 | 6566 |
| 6 | limit 5 to humans | 6396 |
| 7 | limit 6 to English language | 5527 |
| 8 | Limited 7 to ‘‘review’’ | 243 |
