# Supplementary material for: Loneliness and social isolation interventions for older adults: a scoping review of reviews
Source: BMC Public Health. 2020 Feb 14;20:129. doi: 10.1186/s12889-020-8251-6 (PMC7020371; doi:10.1186/s12889-020-8251-6)
Supplement: Supplementary file 2 — Additional file 2: Table S2. Characteristics of reviews included in the scoping review. [file 12889_2020_8251_MOESM2_ESM.docx]

Table 2: Characteristics of literature found on the categorisation of interventions to reduce loneliness and/or social isolation.

Key to study design: RCT, Randomised Controlled Trial.

| **Author(s), year of publication and countries of study (if applicable)** | **Type of review, and inclusion criteria (where applicable)** | **Aims and objectives of review** | **Focus e.g. loneliness, social isolation or both** | **Study designs included, e.g. descriptive or experimental** | **Number of intervention studies and countries delivered where applicable** | **Authors’ categories of intervention(s)** | **Rationale for the categorisation of interventions** |
| --- | --- | --- | --- | --- | --- | --- | --- |
| Rook, 1984  (35) | Literature review. No inclusion criteria | To review existing approaches to reduce loneliness and social isolation and suggest a general framework. | Loneliness and social isolation | Descriptive | Countries were not mentioned in the review | *Goal of Intervention*:  *Facilitate social bonding  *Enhance coping with loneliness  *Prevent loneliness  *Approaches*:  *Individual approaches  *Group approaches  *Environmental approaches | Most of the interventions in these categories fell into four primary strategies of loneliness reduction interventions: improving social skills, enhancing social support, increasing opportunities for social interaction and addressing maladaptive social cognition. Rook stated that interventions for loneliness could have three general goals:   1. Helping lonely individuals establish satisfying interpersonal ties 2. Preventing loneliness from evolving into or contributing to more serious problems i.e. depression, suicide 3. Preventing loneliness from occurring rather than to help people who are already lonely. |
| McWhirter, 1990 (6) | Literature review. No inclusion criteria | To review treatment interventions for loneliness and outline a possible research agenda for further exploration. | Loneliness | Descriptive | Countries were not mentioned in the review | *Cognitive-behavioural therapy  *Social skills training  *Social support | The interventions in the three categories used by the McWhirter (1990) explicitly addresses these four constructs: enhancing social skills, providing social support, increasing opportunities for social interaction and addressing maladaptive social cognition. Similar categorisation as Rook & Peplau (1982) was used. |
| Cattan and White, 1998 (36) | Systematic review.    Studies:   1. Relating to older people; 2. The intervention was intended to target social isolation and/or loneliness; 3. Recording of outcome measures; 4. Interventions that have or intend to achieve health gain; 5. Published in any language, between 1970 and 1997. | To review the effectiveness of health promotion interventions targeting social isolation and loneliness among older people and establish a strategic framework for these programmes. | Loneliness and social isolation | Systematic review identified 21 intervention studies including 10 RCTs and the remainder categorised as either non-RCTs (n=8), before-and-after studies (n=2) or quasi-experimental studies (n=1). | 21 intervention studies identified of which 10 were RCTS.  11 – USA  2 – Canada  2 – Sweden  2 – Denmark  2 – Netherlands  1 – Germany  1 – UK | *Group activity (n=9)  *One to one interventions (n=6)  *Group and one-to-one interventions (n=1)  *Service provision (n=4)  *Whole community approach (n=1) | Interventions were categorised based on an assessment of ‘programme/method type’. |
| Andersson, 1998 (28) | Literature review. No inclusion criteria | To review the scientific efforts concerning social networks with emphasis on loneliness. | Loneliness | Descriptive | Countries were not mentioned in the review | *Social network interventions:*  *Clinical treatment  *Family caretaker enhancement  *Case management  *Neighbourhood helping  *Volunteer linking  *Mutual aid/self-help  *Community empowerment  *Loneliness interventions*  *Goal of interventions:*  Facilitate social bonding  *Individual approaches  - Cognitive behaviour therapy  - Client-centred therapies  - Psychodynamic therapies  *Group approaches  - Social skills training  - Shyness groups  *Environmental approaches  - Network building  - Restructuring social settings  Enhance coping  *Individual approaches  - Improving solitary skills  *Group approaches  - Support groups  Prevention of loneliness  *Group approaches  - Self-help groups (early intervention)  *Environmental approaches  - Community awareness and educational programs  - Removing obstacles to social contact | Interventions were categorised based on a previous review of social network interventions by Biegel (1985). An adaptation of Rook’s (1984) disposition was presented. |
| Findlay, 2003 (37) | Systematic review.  Studies:   1. Published in English between 1982 and 2002; 2. Relating to older people; 3. The intervention was intended to target social isolation and/or loneliness; 4. Recording of outcome measures; 5. Interventions that have or intend to achieve health gain. | To review the effectiveness of interventions that target social isolation amongst older people. | Social isolation | Descriptive and experimental of which 6 of the 17 studies were RCTs. Other study designs included quasi-experimental studies (3), cross-sectional survey (1), non-randomised matched control trial (1), non-randomised post-treatment/test survey (3), observational interview (1) and pre-post intervention study (2). | 17 studies identified  8 – USA  3 – Australia  2 – Canada  2 – Netherlands  1 – Italy  1 – Sweden | *One-to-one (n=5)  *Group Interventions (n=6)  *Service provision (n=2)  *Internet usage (n=4) | Interventions were categorised based on a similar categorisation system by Cattan and White (1998) (e.g. group and one-to-one interventions, service provision and Internet usage). Most of the studies addressed these four constructs: enhancing social skills, providing social support, increasing opportunities for social interaction and addressing maladaptive social cognition. |
| Perese and Wolf, 2005 (38) | Literature review. No inclusion criteria | To provide information to guide nurses in selecting and implementing social network interventions – support groups, psychosocial clubs, self-help groups, mutual help groups and trained volunteers for people with severe mental illness. | Loneliness | Descriptive | Countries were not mentioned in the review | *Social network interventions:*  *Support groups  *Psychosocial clubs  *Self-help groups  *Mutual-help groups  *Trained volunteers | The interventions used by Perese and Wolf (2005) in the categories identified above were similar to those developed for the general population and therefore addressed these constructs: enhancing social skills, providing social support, increasing opportunities for social interaction and addressing maladaptive social cognition. The authors also suggest that whilst all these interventions may address all three of Biegel, et al.’s (1994) domains for reducing social isolation and loneliness (building new network ties, strengthening existing ties and enhancing family ties), they differ in organisational characteristics, effectiveness in reducing loneliness and appropriateness for specific patient groups. |
| Cattan, White, Bond and Learmouth, 2005 (15) | Systematic review.  Studies:   1. Published between 1970 and 2002 in any language; 2. Related to older people; 3. Intervention intended to prevent or alleviate social isolation and/or loneliness; 4. Describing health-promoting interventions that enabled older people to increase control over and to improve their health; 5. Recording of outcome measures. | To review the effectiveness of health promotion interventions that target social isolation and loneliness among older people. | Loneliness and social isolation | Descriptive and experimental with a total of 42 studies. 30 were quantitative outcome studies and the remaining 12 were qualitative observational studies and surveys. 16 of these studies were RCTs and 10 were non-randomised controlled studies. | Of the 30 quantitative outcome studies:  16 – USA  2 – Sweden  3 – Canada  4 – Netherlands  1 – Germany  2 – Denmark  2 - UK | *Group interventions (n=16)  *One-to-one interventions (n=9)  *Both group and one-to-one interventions (n=1)  *Concerning services (n=3)  *Community development (n=1) | Authors used typology from previous review (Cattan and White, 1998) (e.g. group activities, one-to-one counselling, service provision and community development) however categories were further refined in this review. The interventions in the categories used by Cattan, et al. (2005) fell into four primary strategies of loneliness reduction interventions, which are: enhancing social skills, providing social support, increasing opportunities for social interaction and addressing maladaptive social cognition. |
| Oliver, Demiris and Hensel, 2006 (39) | Literature review. No inclusion criteria | To review the potential of videophone technology to improve communication between residents and their family members. | Social isolation | Descriptive | Countries were not mentioned in the review | *Videophone technology | No rationale for categorisation of interventions reported. |
| Grenade and Boldy, 2008 (40) | Literature review. No inclusion criteria | To provide an overview of loneliness and social isolation among older people, including current intervention efforts, addressing both community and residential settings. | Loneliness and social isolation | Descriptive | Countries were not mentioned in the review | *Community settings*  Nature of intervention:  *Group activity based  *One-to-one  *Provision of services  *Community development focus  Goal of intervention:  *Enhancing people’s social networks  *Promoting personal efficacy and behaviour modification and/or skills development  *Residential settings*  Strategies:  *Organised activities to support interaction between residents  *Family friendly policies  *Strategies to maintain links with wider community  *Strategies to support contact with animals | Authors did not provide a rationale for the ‘nature of intervention’. In regards to the ‘goal of intervention’, enhancing people’s social networks was described as facilitating their connection with others whereas behaviour modification and/or skills development was defined as programs to enhance people’s social and/or communication skills. |
| Medical Advisory Secretariat, 2008 (13) | Systematic review.  Studies:   1. English-language reports and human participants; 2. Single-focused interventions directed to or evaluating social isolation or loneliness; 3. Community-dwelling elderly (≥ 65 years) subjects; 4. Quantitative outcome measures on social isolation or loneliness; 5. Study design that included a control or a comparative group. | To review published literature on interventions for social isolation and loneliness in community-dwelling care seniors. | Loneliness and social isolation | 11 quantitative studies were identified of which 6 were RCTs and the balance involved other prospective controlled study designs. Two studies were a form of cluster or community-based intervention. Three nonrandomised studies involved the use of various prospective control groups. | Of the 11 quantitative studies identified:  2 – Sweden  1 – Netherlands  7 – United States  1 - Germany | *In-Person group-based interventions:*  *Support groups (n=4)  *Community-based exercise programs (n=2)  *Technology-assisted intervention for seniors:*  *Telephone support (n=2)  *Hearing loss rehabilitation (n=1)  *Technology-assisted Intervention for caregivers:*  *Nurse-led web-based computer network support (n=1)  *Social worker-led telephone support system (n=1) | No rationale for categorisation of interventions reported. |
| Hawkley and Cacioppo, 2010 (41) | Empirical review. No inclusion criteria | To review the effectiveness of extant interventions to reduce loneliness. | Loneliness | Descriptive | Countries were not mentioned in the review | *Enhancing social skills  *Providing social support  *Increasing opportunities for social interaction  *Addressing maladaptive social cognition | The authors cite that six previously published qualitative reviews (Cattan and White, 1998; Cattan, et al., 2005; Findlay, 2003; McWhirter, B., 1990; Perese and Wolf, 2005; and Rook, 1984) of loneliness intervention literature all explicitly or implicitly addressed the four categories stated above. |
| Masi, Chen, Hawkley and Cacioppo, 2011 (29) | Systematic review and meta-analysis.  Studies:   1. Published in English and in a peer-reviewed journal/doctoral dissertation from 1970 to September 2009; 2. Report original data to avoid inflating effect sizes; 3. Included trials that specifically targeted loneliness among adults, adolescents, and/or children; 4. Intervention directly targets loneliness; 5. Intervention effect had to be measured and reported quantitatively to enable calculation of effect size; 6. Intervention involved a treatment group, not individual cases. | To review the definitions, prevalence, health effects and current theories regarding loneliness, to describe the relationship between these theories and previous studies of loneliness reduction strategies, and to use meta-analytic techniques to quantify the loneliness-reducing effects of studies. | Loneliness | A total of 50 studies of which 12 were single group pre-post studies, 18 non-randomised group comparison studies and 20 randomised group comparison studies. | Countries were not mentioned in the review | *Type of intervention:*  *Social access (n=10)  *Social cognitive training (n=10)  *Social skills training (n=7)  *Social support (n=23)  *Format of intervention:*  *Group (n=33)  *Individual (n=17)  *Mode of intervention*:  *Technology-based (n=15)  *Non-technology (n=35) | Authors did not provide a rationale for the ‘format’ and ‘mode’ categories, however, provided definitions. The rationale used for intervention ‘type’ is that most of the six previous reviews explicitly or implicitly discussed four primary strategies of loneliness reduction interventions (Rook, 1984; McWhirter, 1990; Cattan and White, 1998; Findlay, 2003; Cattan, et al., 2005; Perese and Wolf, 2005). Masi, et al.’s review slightly amended the four primary strategies of loneliness reduction interventions as they used ‘*social access*’ instead of ‘increasing opportunities for social interaction’, additionally, ‘*social cognition training*’ was used instead of ‘addressing maladaptive social cognition’. |
| Dickens, Richards, Greaves and Campbell, 2011 (14) | Systematic review.  Studies:   1. Published in English; 2. Related to older people residing in nursing homes, institutional settings and within the community; 3. The intervention targeted people identified as socially isolated and/or lonely, and stated a clear plausible aim to alleviate this; 4. Recorded participant-level outcome measure, and reported sufficient outcome data for treatment effects to be obtained; 5. Used a randomised controlled trial (RCT), or quasi-experimental (controlled trial or matched controlled trial) design; 6. Included an inactive (usual care, no intervention, attentional) control group. | To review the effectiveness of interventions designed to alleviate social isolation and loneliness in older people and identify potential health benefits of such interventions. | Loneliness and social isolation | Experimental of which 16 were RCTs and 16 were quasi-experimental studies. | Of the 32 studies:  17 – USA  2 – Japan  2 – Sweden  6 – Netherlands  2 – Finland  3 - Canada | *Delivery mode:*  *One-to-one (n=11)  *Group (n=19)  *Mixed mode (n=1)  *Service provision (n=1)  *Intervention type:*  *Offering activities (n=7)  *Offering support (n=15)  *Internet training (n=4)  *Home visiting (n=5)  *Service provision (n=1) | Authors did not provide a rationale for the ‘intervention type’ however implicitly categorised ‘delivery mode’ on the basis of group or one-to-one intervention delivery. |
| Age UK, 2011a (42) | Evidence review. No inclusion criteria | To review the use of technology used by older people. | Loneliness and isolation | Descriptive | Countries were not mentioned in the review | *Modern technology  *Assistive technology | No rationale for categorisation of interventions reported. |
| Age UK, 2011b (43) | Evidence review. No inclusion criteria | To provide evidence to underpin decision-making for people involved in commissioning service development, fundraising and influencing. | Loneliness and isolation | Descriptive | Countries were not mentioned in the review | *One-to-one services  *Group activities  *Community involvement | No rationale for categorisation of interventions reported. |
| Choi, Kong and Jung, 2012 (44) | Systematic review.  Studies:   1. Published in peer-reviewed journals from January 2001 to July 2012 and written in English. 2. Older adults living in either communities or facilities; 3. An intervention that involved computer or Internet use and measured the psychosocial outcomes (i.e., levels of loneliness and depression) of interest. | To examine the effectiveness of computer and Internet training interventions aimed to reduce loneliness and depression in older adults. | Loneliness and depression | Experimental of which 4 are RCTs and 2 are quasi-experimental studies. | Of the 5 studies that presented outcomes on loneliness:  2 – USA  1 – Israel  2 - Netherlands | *Computer and Internet training (n=5) | No rationale for categorisation of interventions reported. |
| Milligan, Dowrick, Payne, Hanratty, Irwin, Neary and Richardson, 2013 (45) | This paper reports the results of two separate systematic reviews:  1) Men’s sheds  2) Other gendered social activity interventions.  Inclusion criteria for both systematic reviews below.  Studies:   1. Published in English, French, Italian and Spanish languages from 1990 to 2013. 2. Described interventions that provide opportunity for older men to come together face to face, in a specified place, for social activities, learning and teaching, or receipt of advice. 3. Study participants were Shed users and their family caregivers, Shed funders and organisers and involved health and social care professionals; 4. Outcomes: considered how the intervention impacts on health, quality of life or wellbeing of participants or their families. | Main aim – to review the effectiveness of Sheds and other gendered social activity interventions for older men. | Loneliness and social isolation | Descriptive | Of the 14 studies for ‘Gendered Social Activity’:  2 – UK  1 – Canada  11 – Australia  Of the 11 studies for the ‘Other gendered interventions for older men’:  4 – Australia  4 – UK  1 – Norway  1 – Canada  1 – Not clearly geographically located | *Gendered social activity  -Sheds (n=14)  -Other gendered interventions for older men (n=11) | No rationale for categorisation of interventions reported. |
| Raymond, Sevigny, Tourigny, Vezina, Verreault and Guilbert, 2013 (46) | Systematic review.  Studies:   1. Population: programmes targeting older people (age criteria as defined in each study); 2. Intervention: programmes supporting or promoting social participation by means of activities maintaining or improving one or several of these issues: social functioning, networking, friendship, social skills, social interactions, social contribution, collective voicing, social roles, social activities, feelings of belonging and inclusion; 3. Comparison: programmes had to have been evaluated by means of a clearly described process of appraisal of a before/after comparison; 4. Outcome: programmes had to evaluate results associated with social participation, as defined in the ‘intervention’ section. | To examine 32 social programmes aimed at fostering the social participation of seniors and propose a typology on each programme’s approach. | Social participation including loneliness and social isolation | Experimental of which 8 are RCTs, 10 were observational studies, 6 were quasi-experimental studies, 5 were pre-test/post-test study designs and 3 were surveys. | Of the 32 studies:  USA – 15  Australia – 2  Canada – 3  China – 1  Finland – 1  Hong Kong – 1  South Africa – 1  UK – 3  Sweden – 3  Netherlands – 1  Japan – 1 | *Social Participation only:*  *Social interaction in an individual context: community-based services programmes (n=1)  *Social interaction in an individual context: at-home context (n=4)  *Social interaction in a group context: classes (n=6)  *Social interaction in a group context: adult day care and senior centres (n=3)  *Collective projects: recreational, sport and socio-cultural activities (n=7)  *Collective projects: intergenerational activities (n=3)  *Volunteering and informal support: organised volunteering (n=4)  *Socio-political involvement and activism: global scope (n=3)  *Socio-political involvement and activism: intergenerational scope (n=1) | The categorisation is embedded in an analysis of the various characteristics of the programmes, namely the type of social situations, interactions and relationships they were enabling or fostering to improve social participation (e.g. a pairing context), as well as the activities they propose to reach these purposes (to track the same example, informal discussion within the pairing context). The purpose of this categorisation is to be used as a convenient and practical tool by all stakeholders interested in the various social programmes available to promote seniors’ social participation. It is not meant to categorise the way such participation is understood. Categories are not intended to be entirely exclusive of one another; overlapping is possible and desirable. |
| Wilson and Cordier, 2013 (47) | Narrative review.  Literature:   1. Between October 2011 and February 2012 relating to community-based Men’s Sheds; 2. No publication date limitations were placed as Men’s Sheds are a recent phenomenon. | To determine the state of the science about the potential for Men’s Sheds to promote male health and well-being. | Social isolation | 22 studies consisting of descriptive designs including descriptive surveys (n=5), mixed methods (n=3) and qualitative interviews (n=4). | Australia | *Adult learning (n=12)  *Health and wellbeing (n=4)  *Meaningful participation (n=3)  *Mentoring (n=1)  *Conceptual framework (n=2) | Literature was categorised into five groups based on either what the authors stated in the studies identified, or the review authors’ interpretation of each paper’s topic of foci. |
| Centre for Policy on Ageing, 2014 (48) | Rapid review. No inclusion criteria | Not reported | Loneliness | Descriptive | Countries were not mentioned in the review | *Information and education  *Group interventions  *Individual interventions:  -Buddying, befriending, companionship, friendship enrichment/assisted friendship  -Companion birds and animals;  -Coping strategies, leisure, mindfulness, meditation and mentoring.  *Regional and environmental interventions  *Telephone based interventions  *Technology-based interventions  *Multi-component interventions  *Interventions in residential care | No rationale for categorisation of interventions and features of some intervention categories were not reported. |
| Centre for Reviews and Dissemination, 2014 (49) | Evidence briefing of systematic reviews.  Literature:   1. Any interventions aimed at reducing or preventing loneliness or social isolation compared to no intervention or usual care; 2. Outcomes of interest include any measures of health services utilisation and associated costs. | To review literature on older people identified as being, or at risk of being, socially isolated and/or lonely. | Loneliness and social isolation | Descriptive of which 7 systematic reviews were identified | Country of interventions not reported | *Group interventions (n=3)  *One-to-one interventions (n=2)  *Technology-assisted interventions (n=5) | No rationale for categorisation of interventions reported and authors stated that there was overlap in terms of scope and included studies in the reviews identified. |
| Hagan, Manktelow, Taylor and Mallet, 2014 (50) | Narrative review.  Studies:   1. Published within years 2000-2012; 2. Peer-reviewed; 3. Published in the English language; 4. With human participants. | To review the effectiveness of social therapeutic interventions to reduce loneliness in older people. | Loneliness | 17 studies identified with experimental designs of which 2 were pilot/exploratory studies, 9 were controlled trial/randomised controlled trials, 3 were evaluation/post-hoc evaluation and 3 were before and after studies. | Of the 17 studies:  USA – 8  UK – 3  Israel – 1  Finland – 1  Holland (Netherlands) – 2  Taiwan – 1  Australia - 1 | *Group, subdivided into: Community-based group intervention and Supported living group intervention (n=8)  *One-to-one (n=3)  *New technologies (n=6) | No rationale for categorisation of interventions reported. |
| Morris, Adair, Ozanne, Kurowski, Miller, Pearce, Santamaria, Long, Ventura, and Said, 2014 (51) | Systematic review.  Studies:   1. Assessed effectiveness of smart technologies on social connectedness (as defined by Thomas, et al. 2010) using some form of intervention study; 2. Published in English and available in full-text from peer review journals; 3. Set in a home environment; 4. Included participants aged ≥45 years. | To examine the effectiveness of smart technologies in improving or maintaining the social connectedness of older people living at home. | Social connectedness including loneliness | 18 studies were included with experimental designs of which 12 were RCTs and the remaining 6 were cohort studies. | Of the 18 studies:  USA – 11  Canada – 1  Netherlands – 4  Norway – 1  Not reported – 1 | *Smart technologies for social connectedness only (n=18) | Loneliness is described as one concept of social connectedness, according to Thomas, et al. (2010). |
| Cacioppo, Grippo, London, Goossens and Cacioppo, 2015 (52) | Literature review. No inclusion criteria | To review the assessment of loneliness and build on the meta-analysis conducted by Masi, et al. (2011) to discuss the efficacy of various treatments for loneliness. | Loneliness | Descriptive | Countries were not mentioned in the review | *One-on-one  *Group therapy  *Wider community interventions  *Models of loneliness interventions:*  *Provide social support  *Increase opportunities for social interaction  *Teach lonely people to master social skills | Authors implicitly addressed the different causes of loneliness and the categorisation of the different ‘models of loneliness interventions’ reflects the intuitive understanding of loneliness. |
| Cohen-Mansfield and Perach, 2015 (53) | Critical review.  Studies:   1. Published between 1996 and 2011; 2. Used a sample of older adults (≥ 55 years of age); 3. Implemented and examined an intervention’s impact on loneliness or identified a situation that directly affected loneliness; 4. Outcome measures include the effects of the intervention or situation on loneliness levels or on loneliness-related measures (e.g. social interactive, social initiative); 5. Pretest-posttest comparisons were made. | To review the utility of loneliness interventions among older persons. | Loneliness | 34 studies included with experimental designs of which 11 were randomised controlled trials, 11 non-randomised controlled trials and 6 non-randomised, non-controlled trials. | Of the 34 studies:  USA – 12  Netherlands – 6  Germany – 1  UK – 2  Finland – 3  Canada – 2  Australia – 2  Sweden – 1  China – 2  Taiwan – 2  Israel – 1 | *Focus of intervention*:  Educational (n=24) subdivided into computerised exercise advisor, psychosocial, occupational therapy and computer training;  Shared Activities (n=6) subdivided into chorale participation, exercise, foster grandparent/psychosocial and visual art discussions;  Sensory technological aids (n=1) (no further subdivision provided);  Activities (n=1) which included only radio programs;  Specific therapy technique (n=2) subdivided into animal assisted and humour therapy.  *Intervention type:*  *Group (n=22)  *One-on-one (n=12) | Authors did not provide a rationale for the ‘intervention type’, however provided definitions for some of the constructs under ‘focus of intervention’. For example, shared activity was defined as an activity that takes place among several persons without an educational or therapeutic context. |
| Davidson and Rossall, 2015 (20) | Evidence review. No inclusion criteria | To summarise available evidence from research on loneliness in later life. | Loneliness | Descriptive | Countries were not mentioned in the review | *Social interaction interventions*:  *Group activities  *One-to-one interventions  *Internet  *Interventions for special groups | Authors only reviewed interventions that tried to increase opportunities for social interaction as this was the only type of interventions delivered in practice in the UK. |
| Elias, Neville and Scott, 2015 (54) | Systematic review.  Studies:   1. Experimental, non-experimental, observational and qualitative designs; 2. Systematic reviews; 3. Population of interest were people aged 60 years and over; 4. Long-term care encompassed nursing homes, assisted living facilities and residential aged care facilities; 5. The intervention was group reminiscence therapy; 6. Outcomes of interest were loneliness, anxiety and depression. | To explore the effectiveness of group reminiscence therapy for older adults with loneliness, anxiety and depression in long-term care. | Loneliness, anxiety and depression | Only 1 study out of 8 had an outcome of loneliness and was a quasi-experimental study. Two studies examined anxiety and five studies examined depression and all eight studies were of a quasi-experimental design. | Taiwan | *Group reminiscence therapy (n=1) | No rationale for categorisation of interventions reported. |
| Franck, Molyneux and Parkinson, 2015 (55) | Systematic review.  Studies:   1. Published as full reports in English; 2. Between January 01, 2009 and December 31, 2013; 3. Reported an intervention that addressed the outcomes of social isolation or loneliness, or the combination of depression with social isolation or loneliness; 4. Involved participants who were mostly aged 60 years and over; 5. Involved participants receiving aged care services (community or residential); 6. Conducted in an urban setting. | To review interventions for reducing social isolation and depression in older people receiving aged care services (community or residential). | Loneliness, social isolation and depression | 5 studies were included of which 2 were experimental designs: 1 quasi-experimental pre- and post-test with control group designs and 2 quasi-experimental pre and post-test designs with no control groups. | Of the 5 studies:  Taiwan – 1  UK – 1  USA – 1  Hong Kong – 1  Australia – 1 | *Group-based activities (n=3)  *Duo-activity intervention (n=1)  *Individual activity (n=1) | No rationale for categorisation of interventions reported. |
| Jopling, 2015 (27) | Narrative synthesis. No inclusion criteria | To reflect the full range of initiatives being undertaken which show promise in tackling loneliness and to examine what hard evidence backs these approaches. | Loneliness | Descriptive | Countries were not mentioned in the review | *Foundation services  *Direct interventions  *Gateway services  *Structural enablers | Interventions were grouped in accordance to addressing these three key challenges:  1)Reaching lonely individuals  2)Understanding the nature of an individual’s loneliness and developing a personalised response  3) Supporting lonely individuals to access appropriate services. |
| Chen and Schulz, 2016 (56) | Systematic review.  Studies:   1. Quantitative and qualitative on the effectiveness of ICT-mediated social isolation interventions for elderly people aged 55 years or older; 2. Published in English between 2002 and 2015. | Objective: To review the effects of Information Communication Technology interventions on reducing social isolation in the elderly. | Social isolation | 30 studies were reported with experimental designs. RCTs comprised 6 studies, another 6 were cohort studies (2 with a control group and 4 without). Of the remaining studies, 4 were cross-sectional studies (surveys) and 14 were qualitative studies: 9 employing in-depth interviews, 3 conducting focus group interviews and 2 applying participation observations. | United States  Austria  Canada  Finland  Israel  Netherlands  New Zealand  Norway  Slovenia  Sweden  Taiwan  UK | *Information Communication Technology interventions (n=30) | No rationale for categorisation of interventions reported. |
| Gardiner, Geldenhuys and Gott, 2016 (16) | Integrative review.  Literature:   1. Relating to interventions with primary or secondary outcomes of reducing or preventing social isolation and/or loneliness; 2. Relating to older adults; 3. Empirical research articles reporting primary research, including all research methodologies (but excluding reviews); 4. English language articles; 5. Published since 2003. | To conduct an integrative review of literature on interventions that target social isolation and/or loneliness in older people. | Loneliness and social isolation | 6 RCTs, 21 quantitative designs, 10 qualitative designs and 2 mixed studies. | Countries were not mentioned in the review | *Social facilitation  *Psychological therapies  *Health and social care provision  *Animal interventions  *Befriending interventions  *Leisure/skills development | Thematic synthesis identified six categories of intervention based on their purpose, their mechanisms of action and their intended outcomes. |
| Poscia, Stojanovic, La Milia, Duplaga, Grysztar, Moscato, Graziano, Collamati, Ricciardi and Magnavita, 2017 (57) | Systematic review.  Studies:   1. Published in English or Italian that implemented loneliness/social isolation interventions; 2. Published between January 2011 and February 2016; 3. Targeted older population; 4. Reported an outcome measure to determine the effects of the intervention for alleviating social isolation or loneliness; 5. Using quantitative or qualitative designs. | To review the effectiveness of existing interventions for alleviating loneliness and social isolation among older persons. | Loneliness and social isolation | Descriptive and experimental of which 4 were RCTs and 6 were pre-post study design. Other included study designs involved pilot trials and quasi-experimental studies. | Quantitative design:  Norway  USA - 3  Japan  Not specified  Australia - 5  Italy  Netherlands  UK - 3  New Zealand  Iran  Qualitative design:  UK - 3  Canada - 2 | *Quantitative designs -*  *Delivery mode:*  *Group (n=11)  *Individual (n=3)  *Mixed (n=4)  *Intervention type:*  *Social support (n=7)  *Social activities (n=2)  *Physical activity (n=3)  *Use of technology (n=4)  *Singing sessions (n=1)  *Horticultural therapy (n=1)  *Qualitative designs* –  *Delivery mode:*  *Group (n=4)  *Individual (n=1) | Authors did not provide a rationale for the ‘intervention type’ however explicitly categorised interventions ‘delivery mode’ on the basis of group or one-to-one intervention delivery. |
| Chipps, Jarvis and Ramlall, 2017 (30) | Systematic review.   1. Participants aged 60 years and above, living in community or residential settings with no major neurocognitive impairments; 2. E-Interventions (interventions that employ any information communication technology (ICT) or Internet-supported intervention delivery mode with or without human support); 3. Quantitative outcome data focussing on social isolation/loneliness; 4. Used systematic review methodology to review quantitative comparative studies. | To synthesise high quality evidence on the effectiveness of e-Interventions to decrease social isolation/loneliness for older people living in community/residential care. | Loneliness and social isolation | 22 e-Interventions primary studies identified. Experimental design of which 6 were RCTs, 8 were quasi-experimental studies and 8 were cross-sectional studies. | USA – 12  Netherlands – 4 | *Online activities (n=15)  *Interpersonal communication (n=1)  *Internet-operated therapeutic software (n=1) | Detailed description of e-Interventions category (already stated in inclusion criteria). The list of primary unique studies was then classified using an adapted Internet-supported interventions classification comprised of three categories of e-Interventions, namely:   1. Online activities (e.g. social media, computer/Internet use and training); 2. Interpersonal communication (e.g. videoconferencing (VC) and Internet-based communication); 3. Internet-operated therapeutic software (e.g. robotics and gaming). |
